# Hybrid Grey Wolf Optimizer with discrete prism dispersion strategy for solving flexible job-shop scheduling problem

**DOI:** 10.1038/s41598-025-33859-x

**Published:** 2026-02-02

**Authors:** Ying Duan, Luyi Shi, Mingyang Li, Kangmin Hua, Ting Liu, Lijun He

**Affiliations:** 1 School of Computer Science, Zhengzhou University of Aeronautics, Zhengzhou, 450046 Henan China; 2 Engineering Research Center of Intelligent Swarm Systems, Ministry of Education, Zhengzhou University, Zhengzhou, 450001 Henan China; 3 Zhengzhou University of Aeronautics, Zhengzhou, 450046 Henan China; 4 Food and Strategic Reserves Administration of Xinjiang Uygur Autonomous Region, Urumqi, 830000 Xinjiang China; 5 School of Transportation and Logistics Engineering, Wuhan University of Technology, Wuhan, 430063 Hubei China

**Keywords:** Engineering, Mathematics and computing

## Abstract

The Flexible job-shop scheduling problem (FJSP) is a quintessential NP-hard problem in the field of production scheduling. With the development of intelligent manufacturing industry, minimizing the total completion time in workshops has become a crucial research focus. Swarm intelligence algorithms have been widely used to solve the FJSP. However, they still suffer from issues such as premature convergence and a tendency of trapping in local optimum. In addition, as iterations increase, the basic parameters of the algorithm still need to be flexibly adjusted. To address these challenges, we propose a hybrid grey wolf optimization algorithm incorporating a discrete prism dispersion strategy (HGWO-DPDS). Inspired by the optical dispersion of light through a prism, this strategy simulates a multi-directional refraction process to diversify the population and improve global exploration. First, in the position update stage, a critical-path-guided mechanism is introduced in the operation sequencing stage to identify and perturb bottleneck operations, while in the machine selection stage, machine-guided convergence enhances the search toward the current best solution. Secondly, the prism-inspired dispersion strategy expands the search directions through multiple reference centers. Finally, an adaptive mutation operator is applied to maintain population diversity and avoid stagnation. We conduct a comprehensive evaluation of the proposed model through benchmark experiments on three widely used datasets—MK, Kacem, and Lawrence instances. HGWO-DPDS is compared with several existing algorithms. The experimental results demonstrate that the proposed framework achieves near-optimal makespan values on most instances, while maintaining stable and reliable performance in solving the FJSP, particularly excelling at escaping local optima compared to existing methods.

## Introduction

The development of Industry 5.0 has brought about a profound transformation in the paradigm of intelligent manufacturing. In contrast to Industry 4.0, which emphasizes large-scale automation and equipment interconnectivity, Industry 5.0 places greater focus on human-centric design and intelligent empowerment. Humans and machines collaborate synergistically to foster the development of more efficient, flexible, and resilient production systems^1^.

In the context of intelligent manufacturing, the FJSP has emerged as a critical bottleneck hindering the improvement of production efficiency, due to its inherent flexibility in both operation routing and machine selection. The primary objective of FJSP is typically to minimize the makespan, which is the maximum completion time of all jobs on their assigned machines. It belongs to the category of NP-hard scheduling problems and is difficult to solve efficiently through traditional precise algorithms^2^.

Swarm intelligence optimization algorithms have become a mainstream method for solving FJSP, owing to their advantages such as strong global exploration capabilities, few control parameters, and good adaptability to complex environments. Among these algorithms, the Grey Wolf Optimizer (GWO) algorithm simulates the leadership hierarchy and hunting behavior of grey wolf groups and performs effectively in continuous space. It has been widely applied to various fields, such as building energy optimization^3^, Internet of Things data transmission^4^ and robotic path planning^5^. However, when dealing with complex and discrete flexible job-shop scheduling environments, the traditional GWO exhibits several limitations, including incompatibility with the discrete search space, premature convergence, and insufficient local escape capability, making it difficult to apply directly.

To address these challenges, this study proposes a hybrid grey wolf optimization algorithm incorporating a discrete prism dispersion strategy (HGWO-DPDS). Unlike existing GWO variants that mainly enhance exploitation through hybrid operators or adaptive weights, the proposed method is the first to draw inspiration from optical physics, introducing a multi-directional dispersion mechanism analogous to light refraction through a prism. This strategy allows multiple reference centers to guide the population toward different regions of the solution space simultaneously, thereby enhancing global exploration and maintaining population diversity.

The proposed HGWO-DPDS constructs a dual-segment encoding and decoding mechanism to adapt the continuous algorithm to the discrete structure of FJSP. In the position update phase, a critical-path-based local perturbation mechanism is employed to refine bottleneck operations that influence the makespan. The discrete prism dispersion strategy generates diverse solutions through a multi-directional refraction process, improving exploration capability. Finally, an adaptive mutation operator dynamically balances exploration and exploitation during the iterative process, enhancing algorithm stability and solution precision. This study not only provides an effective solution for flexible scheduling optimization, but also offers new insights into the application and expansion of swarm intelligence optimization algorithms in Industry 5.0.

The remainder of the paper is organized as follows: Section 2 reviews the related work on the FJSP and the GWO. Section 3 states the research problem of FJSP. Section 4 introduces the basic GWO. Section 5 presents the proposed algorithm HGWO-DPDS, including its structural design and enhancement strategies. Section 6 conducts experiments and analysis on several standard benchmark instances. Section 7 concludes the study and discusses future research directions.

## Related work

### FJSP

Flexible job-shop scheduling is a classical topic in the field of production scheduling. In recent years, metaheuristic algorithms have exhibited a diversified trend of innovation in addressing complex job-shop scheduling problems^6^. Among these, bio-inspired heuristic methods have demonstrated unique advantages in solving FJSP problems by simulating the behavioral search mechanisms of biological populations. By refining the algorithm processes, adjusting key parameters, and introducing novel strategies, the performance of such algorithms can be significantly enhanced, thereby providing effective methodological support for dynamic and adaptive scheduling.

For example, Karaboga proposed the artificial bee colony algorithm (ABC), which outperformed the traditional genetic algorithm and particle swarm optimization (PSO) in numerical optimization tasks^7^. Lin developed a learning-based cuckoo search (LCS) algorithm to address FJSP in semiconductor manufacturing environments^8^. Bharti proposed two hybrid frameworks (Hybrid-I and Hybrid-II) combining PSO, GSA, and GA to solve multi-objective FJSPs, achieving superior performance on benchmark and industrial cases^9^. Park proposed a new unified genetic algorithm, which simplifies the algorithm design and improves the quality of the solution through a sequential coding structure to solve the single-objective flexible job-shop scheduling optimization problem^10^. Kelvin developed a hyper-heuristic algorithm (SA-HH) based on simulated annealing to optimize makespan in high-mix manufacturing scenarios^11^. Fuladi presented a hybrid optimization method that uses genetic algorithm (GA) and simulated annealing (SA) as global optimization techniques, combined with variable neighborhood search, showing promising performance in static and dynamic scheduling contexts^12^. Li studied the flexible job-shop scheduling problem with batch processing (batch flow) and proposed a hybrid algorithm (RL-ABC) that combines reinforcement learning and artificial bee colony to reduce makespan^13^. Zhang proposed a PPO-based deep reinforcement learning framework with graph neural network embedding for the variable processing time FJSP (VPT-FJSP), which outperformed the traditional genetic algorithm and ant colony optimization in static and dynamic scheduling scenarios^14^. Wang developed a dual-attention network (DAN) combined with deep reinforcement learning to capture complex operation-machine relationships in FJSP, achieving results comparable to exact methods and demonstrating strong generalization ability on large-scale and unseen problems^15^.

### GWO

The GWO is a relatively recent meta-heuristic algorithm with a simple structure, easy implementation, and strong adaptability, making it widely applicable in engineering optimization problems.

Mirjalili’s team extended GWO into discrete domains by designing crossover operators and neighborhood search strategies, successfully solving job-shop scheduling problems^16^. Jiang and Zhang proposed a discrete GWO hybridized with Variable Neighborhood Search (VNS) for job-shop and flexible job-shop scheduling problems, and incorporated classical genetic operators while targeting makespan minimization^17,18^. Zhang introduced a crossover mechanism into GWO to address multi-objective FJSP, balancing makespan and machine energy consumption^19^. Lin proposed a learning-based grey wolf optimization algorithm for random flexible job-shop scheduling problems and enhanced resource allocation balance^20^. Kong optimized the makespan and critical machine load of FJSP, achieving better in terms of solution accuracy and convergence performance^21^. Zhang integrated Q-learning into GWO to develop low-carbon and efficient scheduling strategies for complex manufacturing systems^22^. Zhu addressed FJSP with job priority constraints and proposed the self-adaptive cell evolution-based grey wolf optimizer (SCEGWO) to minimize completion time^23^. Chen focused on the distributed hybrid flow shop scheduling problem and proposed a Q-learning-based grey wolf optimization algorithm considering practical constraints^24^. Li incorporated the demands of green intelligent manufacturing and constructed a dynamic flexible job-shop multi-objective scheduling model, considering criteria such as energy consumption, makespan, machine load, and product quality stability. He also proposed an Improved Multi-objective GWO (IMOGWO) to solve the model^25^. Zhou proposed an adaptive fast GWO (SS-GWO) to solve FJSP, achieving better optimization results and lower makespan compared to existing algorithms^26^.

### Main contributions of this paper

The aforementioned research on GWO has achieved notable progress in the field of flexible workshop job scheduling. However, the algorithm still suffers from limited global search capability and is prone to premature convergence, making it susceptible to being trapped in a local optimum. Therefore, further systematic research is needed to optimize and extend the algorithm and to develop more efficient strategies for practical scheduling. This paper proposes a hybrid grey wolf optimization algorithm HGWO-DPDS, designed to enhance the population diversity and reduce the makespan.

The main contributions of this study are summarized as follows:

1. The nonlinear convergence factor *a* is introduced to improve the convergence characteristics of the algorithm.
2. A discrete prism dispersion strategy (DPDS), inspired by the optical dispersion phenomenon, is proposed for the first time in contrast to existing algorithms. By generating multiple reference centers and dispersing individuals toward them, this mechanism enables multi-directional exploration and enhances population diversity.
3. In the position update phase, two local perturbation strategies are designed for the bottleneck operations on the critical path machine to improve the local refinement accuracy. In addition, for the machine selection segment, a “three-wolf voting” with roulette-based probability selection is adopted to move toward one of the three leading wolves with a certain probability.
4. A mutation operator with a dynamic adaptive attenuation mechanism is integrated to adjust the mutation rate over time. This enhances the fine-tuning ability of the solution in the middle and late stages of the iteration, and improves algorithm stability and solution accuracy.

## Problem description

In a flexible workshop environment, there are *n* jobs and *m* machines. Each job consists of a sequence of operations that must be processed in a predefined order. Each operation $O_{ij}$ can be executed on one of several available machines $M \subseteq M_{ij}$ and the processing time may vary depending on the machine selected. Each machine can process only one operation at any given time, and once the processing begins, it cannot be interrupted. The scheduling must strictly follow the technological precedence constraints, meaning that operation $O_{ij}$ cannot begin until its immediate predecessor $O_{i(j-1)}$ has completed. Based on the above analysis, FJSP can be decomposed into two interrelated sub-problems:

1. *Machine allocation problem*: Select the appropriate machine from the eligible set for each operation.
2. *Operation sequencing problem*: Determine the processing order of the operations on each machine.

The symbols, abbreviations, and algorithm parameters employed in this study are listed in Tables 1 and 2.

**Table 1:** Symbols and abbreviations used in this study.

Symbols	Description
*n*	Total number of jobs
$n_{op}$	Total number of operations
*m*	Total number of machines
*h*	Machine index
*i*	Job index
$j_{i}$	Total number of operations in the $i^{th}$ job
*L*	A sufficiently large positive number
*J*	The set of jobs, $J = \{J_1, J_2,..., J_n\}$
$O_{ij}$	The $j^{th}$ operation of the $i^{th}$ job
$M_{ij}$	Machine set that can process $O_{ij}$
$P_{hij}$	Processing time of $O_{ij}$ on machine *h*
$s_{ij}$	Start time of the $j^{th}$ operation of the $i^{th}$ job
$c_{ij}$	End time of the $j^{th}$ operation of the $i^{th}$ job
$C_{\max }$	Makespan
$C_i$	Completion time of job *i*
$X_i$	Solution individual
$x_{hij}$	Binary decision variable, equals 1 if operation $O_{ij}$ of job *i* is processed on machine $M_{h}$, 0 otherwise
$y_{hijkl}$	Binary decision variable, equals 1 if operation $O_{hij}$ precedes operation $O_{hkl}$,0 otherwise
*i*	is processed before operation $O_{hkl}$ on machine $M_{h}$, 0 otherwise
MS	Machine selection encoding
OS	Operation sequence encoding

**Table 2:** Algorithm parameters used in this study.

Parameter	Description
*a*	The convergence factor
*N*	Population size
*T*	Maximum number of iterations
*t*	Current iteration
*k*	Refraction parameter
*r*	Dispersion probability
$\mu _0$	Initial mutation intensity
$\mu _{\min }$	Minimum mutation intensity
*iter*	The current number of iterations
*S*	The machine schedule matrix

The optimization objective is to minimize the makespan ($C_{\max }$). The mathematical formulation is given as:

1 $$\begin{aligned} \min C_{\max } = \min \left( \max _{1 \le i \le n} C_i\right) \end{aligned}$$

where $C_{i}$ is the completion time of the last operation in $J_{i}$. The model is subject to the following constraints^2^:

1. Operation timing and precedence constraint:
  2 $$\begin{aligned} & s_{ij} + x_{hij} \cdot P_{hij} \le c_{ij}, \quad i = 1,2,\dots ,n;\ j = 1,2,\dots , j_i;\ h = 1,2,\dots ,m \end{aligned}$$
  3 $$\begin{aligned} & c_{ij} \le s _{i(j+1)}, \quad i = 1,2,\dots ,n;\ j = 1,2,\dots , j_i - 1;\ h = 1,2,\dots ,m \end{aligned}$$
  These constraints ensure that each operation is completed after its processing time elapses, and that the subsequent operation in the same job starts only after its predecessor is completed.
2. Makespan constraint :
  4 $$\begin{aligned} c_{ij} \le C_{\max }, \quad \forall i, j \end{aligned}$$
  This constraint ensures that the completion time of each job does not exceed the makespan.
3. Operation completion time definition:
  5 $$\begin{aligned} c_{ij} = s_{ij} + \sum _{h \in M_{ij}} x_{hij} \cdot P_{hij}, \quad i = 1,2,\dots ,n;\ j = 1,2,\dots ,j_i;\ h = 1,2,\dots ,m \end{aligned}$$
  The completion time of an operation is determined by its start time and the processing time on the selected machine.
4. Disjunctive and Precedence Constraints:
  6 $$\begin{aligned} s_{ij} + P_{hij}&\le s_{kl} + L(1 - y_{hijkl}), \nonumber \\&\quad \begin{aligned}&i = 1,2,\dots ,n; \quad j = 1,2,\dots ,j_i; \quad h = 1,2,\dots ,m; \\&k = 1,2,\dots ,n; \quad l = 1,2,\dots ,j_i \end{aligned} \end{aligned}$$
  7 $$\begin{aligned} c_{ij}&\le s_{i(j+1)} + L(1 - y_{hklj(i+1)}), \nonumber \\&\quad \begin{aligned}&i = 1,2,\dots ,n; \quad j = 1,2,\dots ,j_i - 1; \quad h = 1,2,\dots ,m; \\&k = 1,2,\dots ,n; \quad l = 1,2,\dots ,j_i \end{aligned} \end{aligned}$$
  Specifically, the first constraint ensures that two operations assigned to the same machine are scheduled without time overlap. The second constraint enforces the precedence relationship within each job, requiring that each operation must be completed before its subsequent operation starts.
5. Machine assignment constraint:
  8 $$\begin{aligned} \sum _{h=1}^{m_{ij}} x_{hij} = 1, \quad i = 1,2,\dots ,n;\ j = 1,2,\dots ,j_i;\ h = 1,2,\dots ,m \end{aligned}$$
  where $x_{hij} = 1$ if operation $O_{ij}$ is assigned to machine $M \subseteq M_{ij}$, otherwise $x_{hij} = 0$. The machine assignment variable $x_{hij}$ is binary: it equals 1 if operation $O_{ij}$ is assigned to machine $M_{k}$ and 0 otherwise. This enforces exclusivity in the assignment decision.
6. Positive timing constraint:
  9 $$\begin{aligned} s_{ij} \ge 0, \quad c_{ij} \ge 0, \quad i = 1,2,\dots ,n;\ j = 1,2,\dots ,j_i \end{aligned}$$

Each operation must have non-negative start and completion times, ensuring physical feasibility of the schedule.

## Basic GWO

The GWO algorithm is used to simulate the social hierarchy and hunting behavior of grey wolf groups in nature^16^, such as tracking and attacking prey. It achieves a balance between global and local exploration in the solution space and has been widely applied in complex optimization problems, such as job-shop scheduling, path planning, machine learning, and image processing.

In GWO, each individual in the group represents a grey wolf, divided into four hierarchical roles: $\alpha$ (optimal solution), $\beta$ (suboptimal solution), $\delta$ (the third best solution) and $\omega$ (the remaining individuals). During each iteration, the position update of individuals is guided primarily by the first three, enabling the population to gradually converge toward the optimal solution.

### Mathematical model description of grey wolf hunting behavior

10 $$\begin{aligned} & \vec {D} = \left| \vec {C} \cdot \vec {X}_p(t) - \vec {X}(t) \right| \end{aligned}$$

11 $$\begin{aligned} & \vec {X}(t+1) = \vec {X}_p(t) - \vec {A} \cdot \vec {D} \end{aligned}$$

where $\vec {X}_p(t)$ denotes the position vector of the prey, $\vec {X}(t)$ is the current position vector of the grey wolf, and $\vec {A}$, $\vec {C}$ are the coefficient vectors. The distance vector $\vec {D}$ represents the distance between an individual and the prey.

The position update of each individual is influenced by coefficient vectors that balance exploration and exploitation. The two main coefficient vectors $\vec {A}$ and $\vec {C}$ are defined as:

12 $$\begin{aligned} & \vec {A}=2\cdot \vec {a}\cdot \vec {r}_1-\vec {a} \end{aligned}$$

13 $$\begin{aligned} & \vec {C}=2\cdot \vec {r}_{2} \end{aligned}$$

where $\vec {a}$ is a control vector that decreases from 2 to 0 with the number of iterations and is used to adjust the search range, while $\vec {r}_1$ and $\vec {r}_{2}$ are random vectors in the interval [0, 1].

### Leader-based position update

To improve the quality of the solution, GWO utilizes the positions of the top three leaders: $\alpha$, $\beta$, and $\delta$ to guide each individual in the population:

14 $$\begin{aligned} & \vec {D}_\alpha =\left| \vec {C}_1\cdot \vec {X}_\alpha -\vec {X}\right| \end{aligned}$$

15 $$\begin{aligned} & \vec {D}_\beta =\left| \vec {C}_2\cdot \vec {X}_\beta -\vec {X}\right| \end{aligned}$$

16 $$\begin{aligned} & \vec {D}_\delta =\left| \vec {C}_3\cdot \vec {X}_\delta -\vec {X}\right| \end{aligned}$$

where $\vec {D}_\alpha$ , $\vec {D}_\beta$ and $\vec {D}_\delta$ can be interpreted as the distance vectors between the current individual and the position of three leading wolves, which represent the relative position of the leader wolf and the current individual, thus generating a search or update direction. $\vec {X}_\alpha$ , $\vec {X}_\beta$ and $\vec {X}_\delta$ represent the positions of the leadership individuals $\alpha$ , $\beta$ and $\delta$ in the $t^{th}$ iteration respectively. Each leading wolf is associated with an independent coefficient vector $\vec {C}_i$ to ensure diversity in the update directions.

17 $$\begin{aligned} & \vec {X}_1=\vec {X}_\alpha -\vec {A}_1\cdot \vec {D}_\alpha \end{aligned}$$

18 $$\begin{aligned} & \vec {X}_2=\vec {X}_\beta -\vec {A}_2\cdot \vec {D}_\beta \end{aligned}$$

19 $$\begin{aligned} & \vec {X}_3=\vec {X}_\delta -\vec {A}_3\cdot \vec {D}_\delta \end{aligned}$$

20 $$\begin{aligned} & \vec {X}(t+1)=\frac{1}{3}\left( \vec {X}_1+\vec {X}_2+\vec {X}_3\right) \end{aligned}$$

where $\vec {X}(t+1)$ is used to update the positions of ordinary individuals, which is equivalent to taking the average of the three candidate solutions $\vec {X}_1$, $\vec {X}_2$ and $\vec {X}_3$ based on the guidance of the three leaders relative to the current individual. This process reflects a weighted consensus approach that enables the population to generate the next generation of individuals under the guidance of the top three wolves.

The parameter $\vec {A}_i$ is a control parameter that adjusts the tendency toward local exploitation or global exploration. Usually, when $\vec {A}_i$ has a larger value, the step size increases accordingly, allowing the algorithm to explore new regions of the solution space. Conversely, when $\vec {A}_i$ is smaller, the update becomes more fine-grained, favoring local convergence.

This mechanism reflects the guiding role of social hierarchy in the search process, prompting the population to converge to the optimal solution while retaining sufficient diversity to avoid premature convergence.

## The Hybrid Grey Wolf Algorithm HGWO-DPDS

### Overview of the Hybrid Grey Wolf Algorithm

The improved algorithm proposed in this study, referred to as HGWO-DPDS, mainly consists of five stages: initialization, encoding, fitness evaluation, position updating, and mutation operation. The algorithm aims to minimize the makespan of FJSP. The detailed procedure is outlined in Algorithm 1:

**Algorithm 1:**
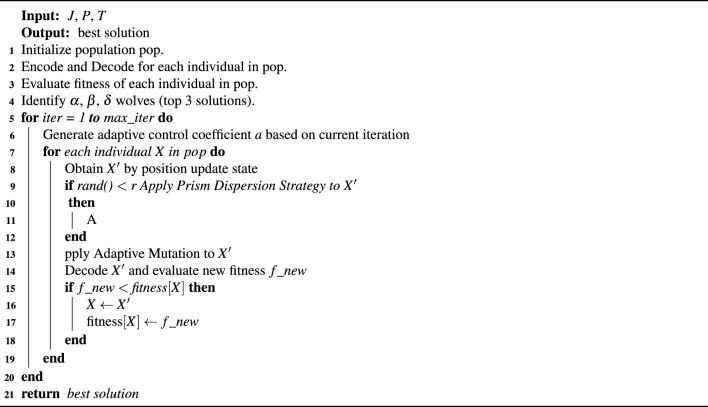
HGWO-DPDS

It integrates several enhancement strategies into the standard Grey Wolf Optimizer to improve its efficiency in solving the FJSP. Specifically, the initialization phase generates a set of candidate solutions to enhance global exploration, while the encoding stage ensures that each individual can be accurately mapped to a feasible schedule. During the fitness evaluation, the makespan of each individual is computed through a decoding mechanism under the FJSP constraints. The position update incorporates a critical-path-based mechanism that perturbs bottleneck operations to enhance exploration, while a leader-machine-guided strategy directs convergence toward high-quality solutions. In addition, the discrete prism dispersion strategy diversifies the population by guiding solutions toward multiple reference centers, and the adaptive mutation further refines promising solutions to prevent premature convergence.

### Discrete dual-segment encoding and decoding strategy

This study uses three effective bio-inspired heuristic strategies to initialize the population and construct approximate solutions, with an initialization ratio of 1:1:2.

1. High-fitness clustering: Based on the principle of minimizing the overall machine load, the most suitable machines are assigned to all operations;
2. Local cross sampling: Within the optional machine set of a single job, the local greedy rule is applied to generate fused solutions;
3. Random population embedding: Random sampling within the legal machine set of each operation. The three strategies generate a set of individuals with different scheduling codes. This hybrid initialization method balances the diversity and quality of the solutions at an early stage, providing a solid foundation for subsequent search.

Encoding is the expression of individual solutions. An efficient encoding scheme must ensure the validity and feasibility of individuals. Traditional GWO mainly targets continuous optimization problems, while FJSP belongs to the category of discrete resource allocation problems. Therefore, it is essential to develop a proper encoding and decoding strategy.

We use a discrete dual-segment encoding to represent each solution. The chromosome is composed of the *machine selection segment* (MS) and the *operation sequencing segment* (OS).

Each element of the MS is an integer-coded representing the relative index of the current operation in its optional machine set, and the OS is the sequence of the job numbers during scheduling, with each job number appears as many times as its operations. Repeated occurrences of a job index are mapped to its operations in order: the first appearance of job i corresponds to its first operation, the second appearance to its second operation, until all operations of the job are assigned.

Moreover, invalid encodings generated during solution updates are repaired through a two-stage procedure. The OS part is corrected by adjusting the number of occurrences of each job to match its required operations. The MS part is repaired by replacing any out-of-range machine index with a feasible one according to each candidate machine set.. This ensures that all individuals remain feasible.

The complete representation of a scheduling solution is as follows:

21 $$\begin{aligned} \text {Individual} = \underbrace{[ms_1, ms_2, \ldots , ms_{n_{op}}]}_{\text {Machine selection segment}} \Vert \underbrace{[os_1, os_2, \ldots , os_{n_{op}}]}_{\text {Operation sequencing segment}} \end{aligned}$$

The detailed decoding framework is outlined in Algorithm 2:

**Algorithm 2:**
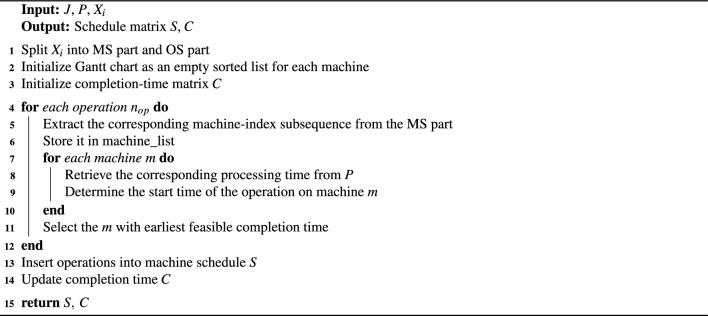
Decoding

To clearly illustrate this decoding method obviously, a problem with four jobs, three machines, and ten operations is provided as an example. This instance of FJSP is shown in Table 3.

**Table 3:** An example of FJSP.

Jobs	Operations	Machines		
		$M_1$	$M_2$	$M_3$
$J_1$	$O_{11}$	6	$\infty$	5
	$O_{12}$	$\infty$	7	4
$J_2$	$O_{21}$	9	6	7
	$O_{22}$	5	$\infty$	6
	$O_{23}$	5	6	$\infty$
$J_3$	$O_{31}$	$\infty$	5	6
	$O_{32}$	7	4	$\infty$
$J_4$	$O_{41}$	$\infty$	8	6
	$O_{42}$	4	5	3
	$O_{43}$	6	7	$\infty$

**Fig. 1:**
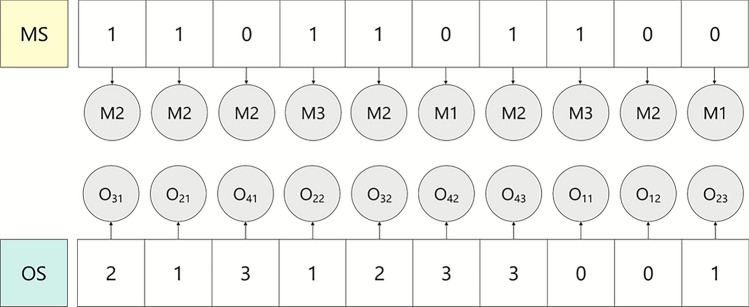
The encoding and decoding of an individual solution.

**Fig. 2:**
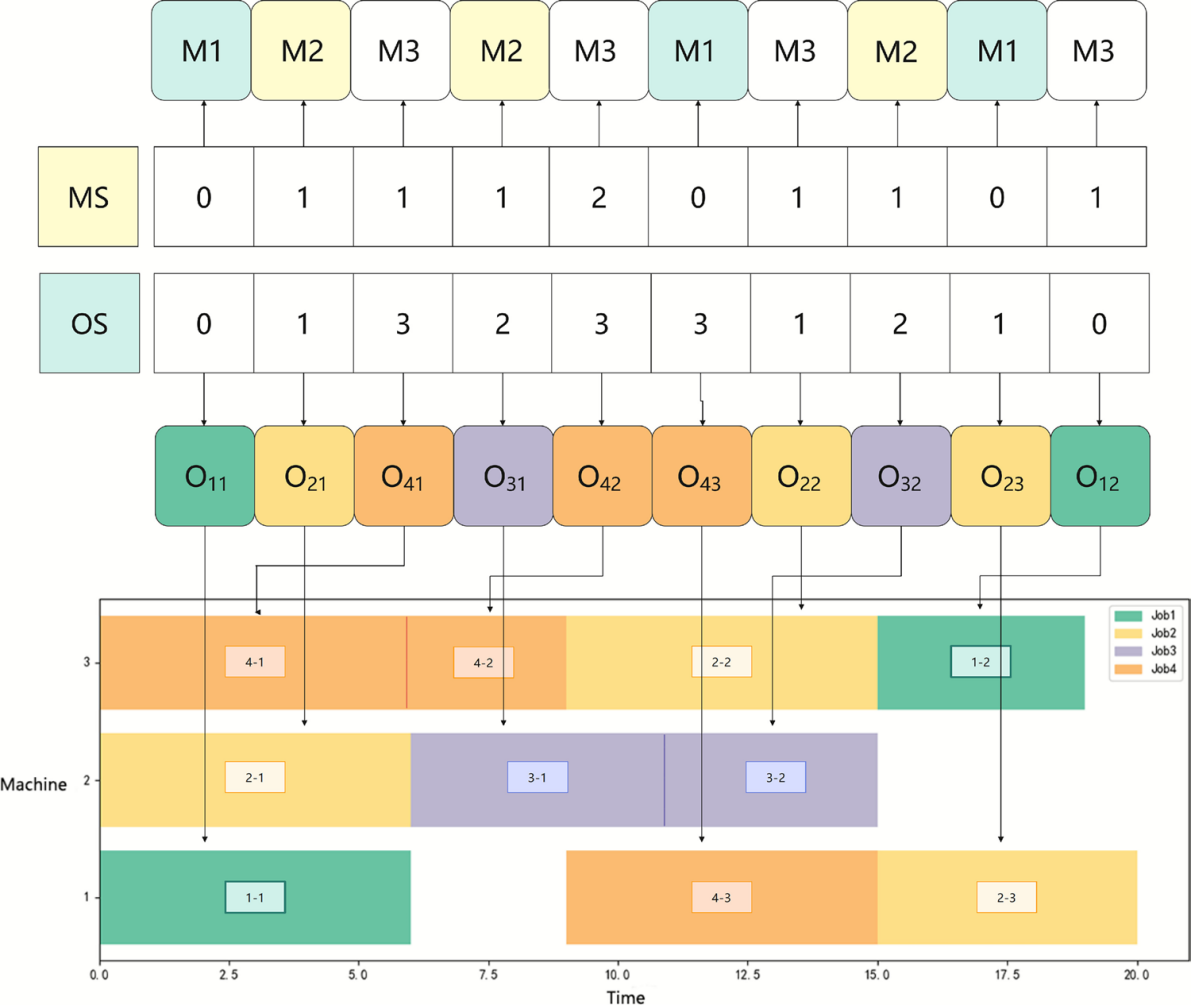
Scheduling Gantt chart.

A feasible encoding can be randomly generated as follows, where the index starting from 0. The encoding and decoding process is illustrated in Fig. 1, and the best solution obtained by executing the algorithm is shown in Fig. 2.

As shown in Fig. 2, for the MS, the first gene 1 indicates that the first operation $O_{31}$ of the job 3 is processed at index 2 of its optional machine set, which is machine $M_{2}$; similarly, the second 1 indicates that the second operation $O_{21}$ of the job 2 is assigned to machine $M_{2}$.

In the OS, each job number indicates the next operation of that job to be scheduled. The first gene 2 represents the first operation $O_{31}$ of job 3, and the second gene 1 represents the operation $O_{21}$ of job 2, and so on. The complete scheduling scheme can be decoded, and the corresponding Gantt chart obtained accordingly.

Specifically, the operation sequence on machine $M_{1}$ is $O_{11} \rightarrow O_{43} \rightarrow O_{23}$, the operation sequence on machine $M_{2}$ is $O_{21} \rightarrow O_{31} \rightarrow O_{32}$, and the operation sequence on machine $M_{3}$ is $O_{41} \rightarrow O_{42} \rightarrow O_{22} \rightarrow O_{12}$.

### Discrete prism dispersion strategy

The physical essence of light dispersion comes from the law that the refractive index of a material varies with wavelength of incident light. According to Snell’s law^27^, also known as the law of refraction:

22 $$\begin{aligned} n_{1}\sin \theta _{1}=n_{2}(\lambda )\sin \theta _{2} \end{aligned}$$

where $n_{1}$ represents the refractive index of the incident medium, $n_{2}(\lambda )$ represents the refractive index of the refractive medium at the wavelength $\lambda$, and $\theta _{1}$, $\theta _{2}$ are the angles between the incident light, the refracted light and the interface normal respectively.

**Fig. 3:**
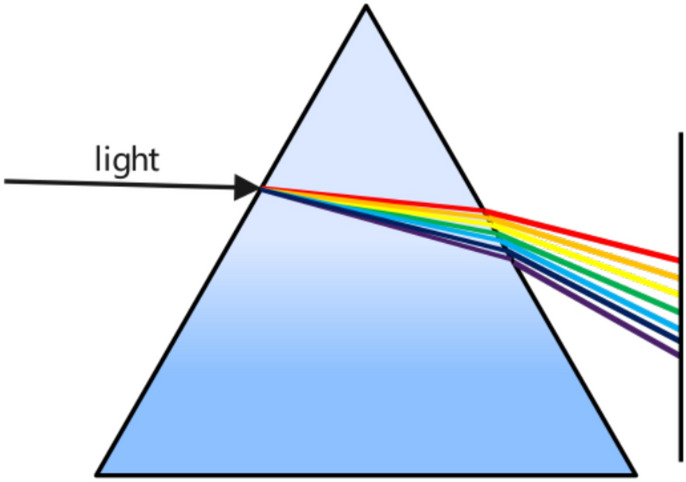
Prism dispersion of light.

Figure 3 illustrates the physical process of light dispersion through a prism, in which incident white light is decomposed into multiple colored rays due to wavelength-dependent refraction. When light enters the prism from air, the violet component will be deflected more due to its higher refractive index, while the red light will be deflected less. As a result, the mixed white light is decomposed into a series of orderly arranged colored light bands, forming a typical spectral dispersion phenomenon.

In the later stage of the traditional grey wolf optimization algorithm, most individuals tend to cluster around the best solution $\alpha$, leading to rapid convergence, which also results in a sharp decline in population diversity, thereby weakening the global exploration ability. Although there is a certain guiding relationship between individuals and superior wolves, its essence remains a one-directional, nearest-neighbor convergence process, lacking an effective mechanism for escaping local optimum.

**Fig. 4:**
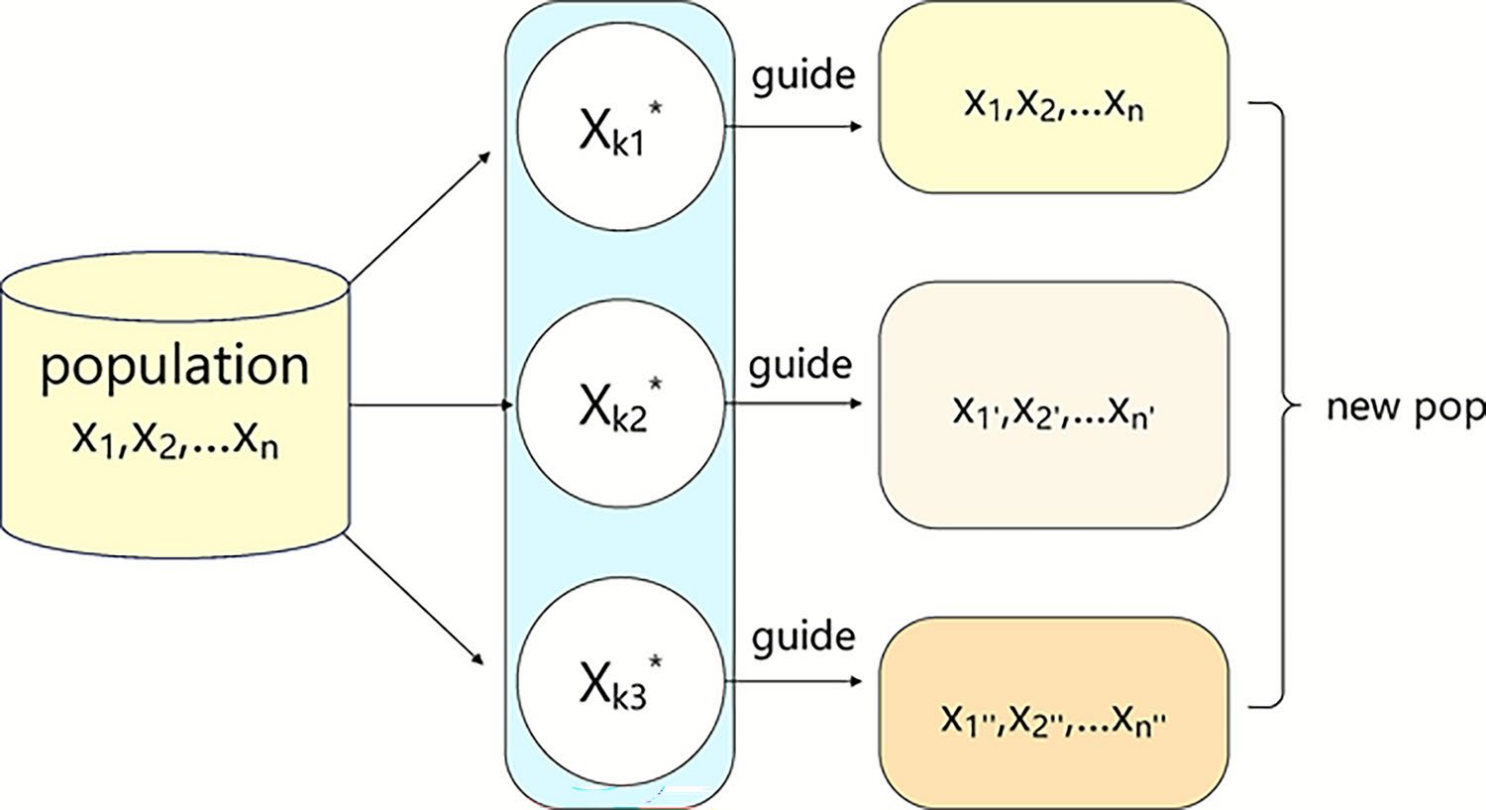
Execution process of the DPDS.

From the perspective of heuristic optimization, the prismatic dispersion of light illustrates that a simple system produces a wide range of diverse outcomes after interacting with a complex medium, which can be likened to a multi-directional perturbation strategy. This study simulates the dispersion behavior of light, drives population diversification in multiple directions, enhances the exploration capability in the middle and late stages of algorithm, and avoids falling into the local optimum. The detailed process of DPDS is outlined in Algorithm 3, and the execution process is illustrated in Fig. 4.

**Algorithm 3:**
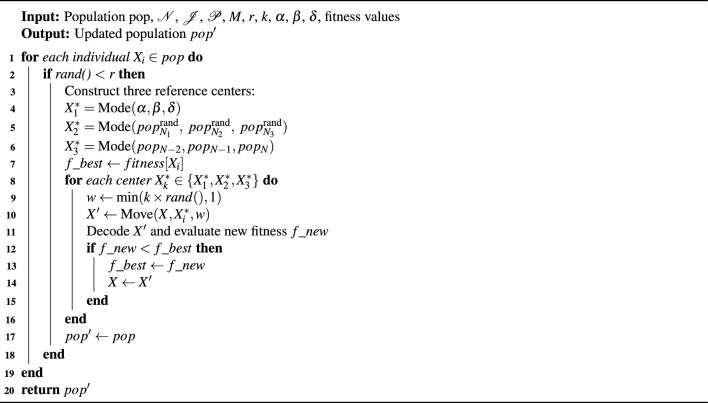
DPDS

The current individual is represented as $x_{i}$, corresponding to a feasible scheduling solution. We introduce several reference centers $x_k^*$, each constructed by taking the majority vote on the MS and OS segments among three selected reference individuals, where for each gene position, the value that appears most frequently among the three individuals is retained. If all three values differ, one of them is randomly selected. The individuals are selected using three strategies: (1) the three best solutions ($\alpha$, $\beta$, and $\delta$), (2) three randomly chosen individuals from the population, and (3) the last three individuals in the population.

**Fig. 5:**
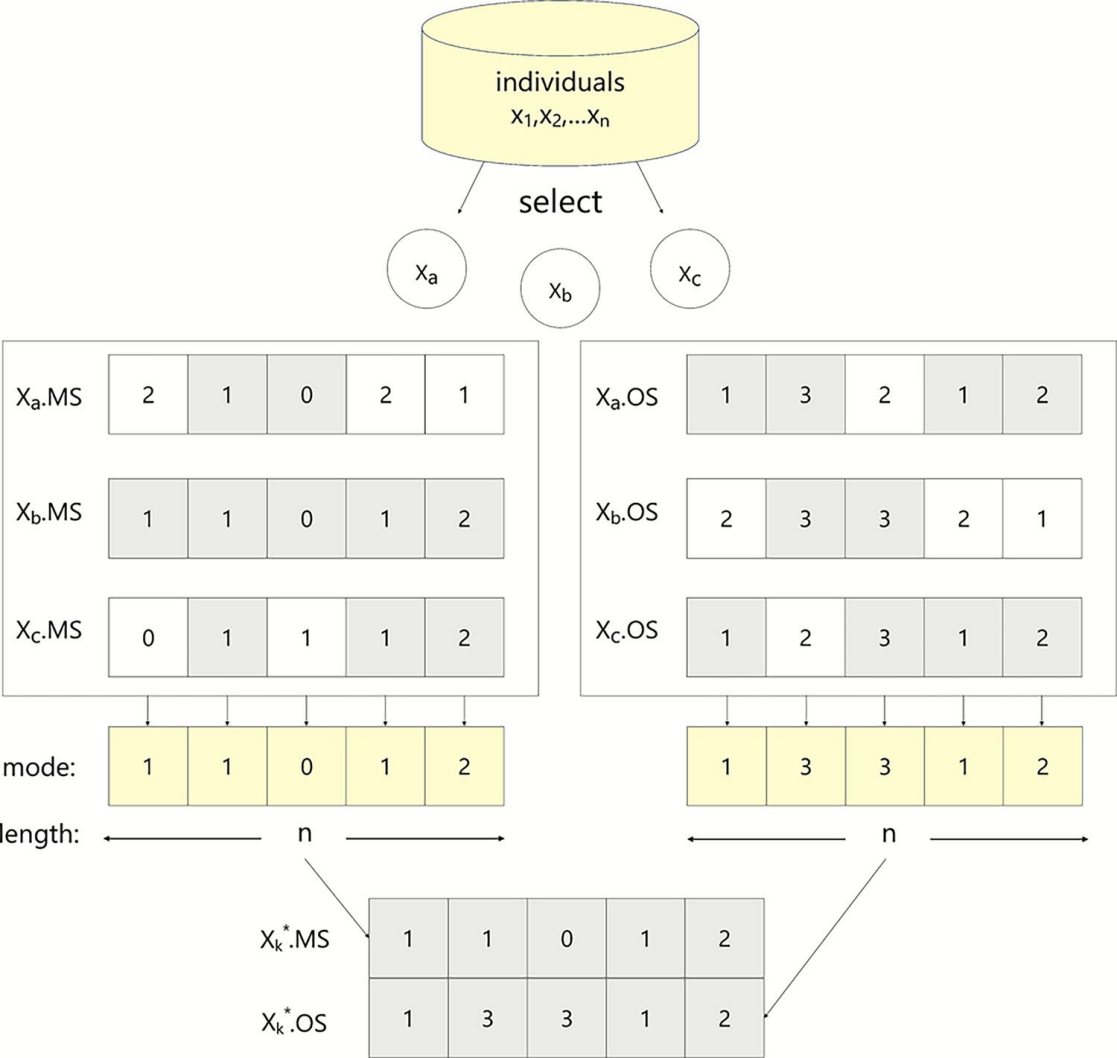
The construction process of a reference center.

Equation (25) indicates that the center is obtained through majority voting of three reference individuals, and the construction process of the reference centers based on the MS and OS segments is illustrated in Fig. 5.

23 $$\begin{aligned} x_k^*=\operatorname {Mode}\left( x_{a}, x_{b}, x_{c}\right) \end{aligned}$$

This process is analogous to the formation of a multi-color dispersion center. For each gene of a candidate solution, it moves toward the corresponding reference center with probability $x \in [0,1]$. The mathematical model is defined as follows:

24 $$\begin{aligned} x_{i, j}^{\text {new}}=\left\{ \begin{array}{cl} x_{k,j}^*,\text { if}& x_{i, j}\ne x_k^*\text { and}\text { x}<w\\ & x_{i, j},\text { otherwise} \end{array}\text { for} j=1,\ldots , 2 n\right. \end{aligned}$$

where $x_{k,j}^*$ denotes the value of the reference center individual at the $j^{th}$ gene position. Assume that both the MS and OS segments have a length of *n*. If $j < n$, the $x_{i,j}$ represents the machine selection index of the $j^{th}$ gene position of the $i^{th}$ individual; otherwise, it represents the operation sequencing index to be scheduled at the $(j-n)^{th}$ gene position. The probability *x* is determined by the perturbation strength *w*, which is generated based on refractive intensity factor *k* and a random number drawn from a uniform distribution. A larger value of *k* increases the likelihood of perturbation. The expression is as follows:

25 $$\begin{aligned} w=\min (k\cdot \operatorname {rand}(), 1) \end{aligned}$$

Although DPDS is inspired by optical dispersion, it is conceptually related to center-guided recombination mechanisms such as multi-parent crossover^28^ and EDA-based model learning^29^. Unlike these methods, which depend on stochastic gene mixing or probabilistic sampling, DPDS generates multiple projections around solutions, providing structured and diversity-enhancing perturbations while avoiding randomness-induced drift common in other approaches. These make DPDS a stable mechanism.

### Position update

Position update is the core mechanism of GWO. In the standard GWO, the position of each individual approach the high-quality solution area through a weighted average. This mechanism ensures strong local exploration capability and fast convergence.

However, directly applying this update rule to FJSP may be ineffective: first, it is based on real number operations and cannot manipulate the discrete coding structure; second, its rapid convergence often causes premature stagnation. Therefore, we develop a customized position update strategy that is adapted to the FJSP and introduces a local search strategy to enhance search diversity. The position update procedure is denoted as follows:

Step 1: Given the current individual chromosome, decode it to identify the critical machine that contributes most to the makespan, then extract the scheduling block of this machine.

Step 2: If the sub-block contains at least two operations, apply a local search strategy (selected from two available strategies) to update the critical block’s operation sequence. Sub-blocks shorter than 2 contain only a single operation and moving it would have negligible impact on the makespan, whereas longer sub-blocks allow meaningful local perturbations.

Step 3: Obtain the machine selection segments from the three leading individuals, and update the machine selection of the current individual based on one of the leading wolves.

Step 4: Return the updated individual chromosome as the new solution.

The local search strategy used in this study consists of two components: operation sequencing and machine selection.

**Fig. 6:**
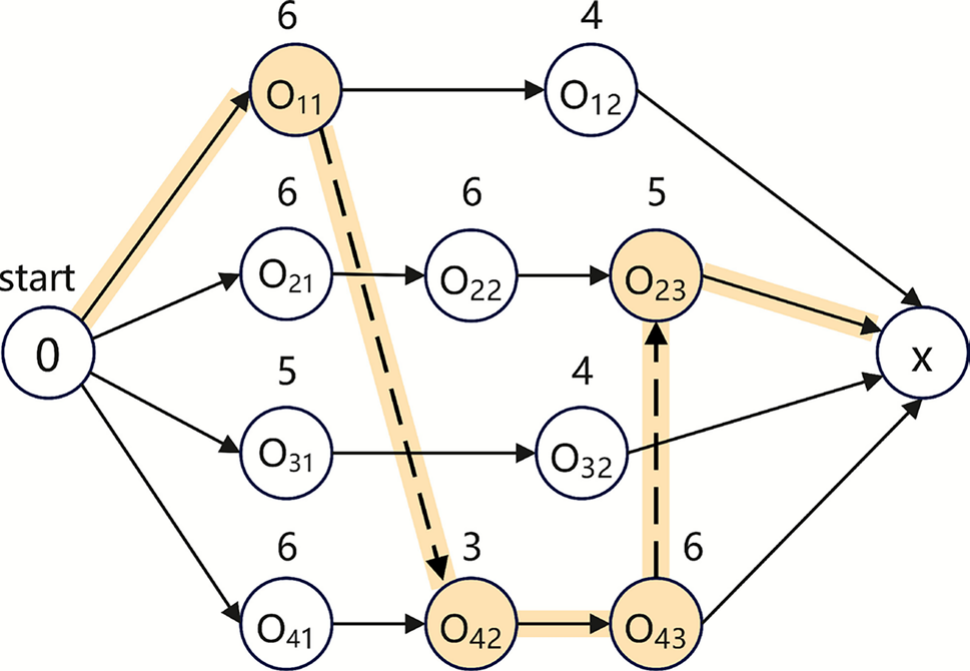
Critical path in the disjunctive graph model.

For the operation sequence, a search mechanism guided by the fusion of local critical paths is employed. In scheduling problem, the critical path refers to a series of sequential operations that determine the makespan. It can be considered as a set of serially dependent operations with the following characteristics: (1) It starts from the first operation of the longest time-consuming route and ends at the last operation, which is a complete path; (2) Each operation on this path must wait either for its predecessor to complete or for the assigned machine to become available; (3) The total duration of all operations in the critical path equals the makespan of the schedule; (4) Any delay in an operation on the critical path will directly lead to an increase in the overall makespan. The processing order and resource conflict along the critical path affect the scheduling efficiency and overall performance (Fig. 6).

Based on this observation, applying local perturbations to the critical path can effectively optimize the overall makespan.

For each individual entering the position update phase, the processing status on each machine is decoded, and the completion time of the last operation on each machine is recorded. The machine with the longest completion time is identified as the critical machine. The corresponding sequence of operations on this machine is defined as the sub-block of the critical path, representing a potential bottleneck affecting the overall makespan.

**Fig. 7:**
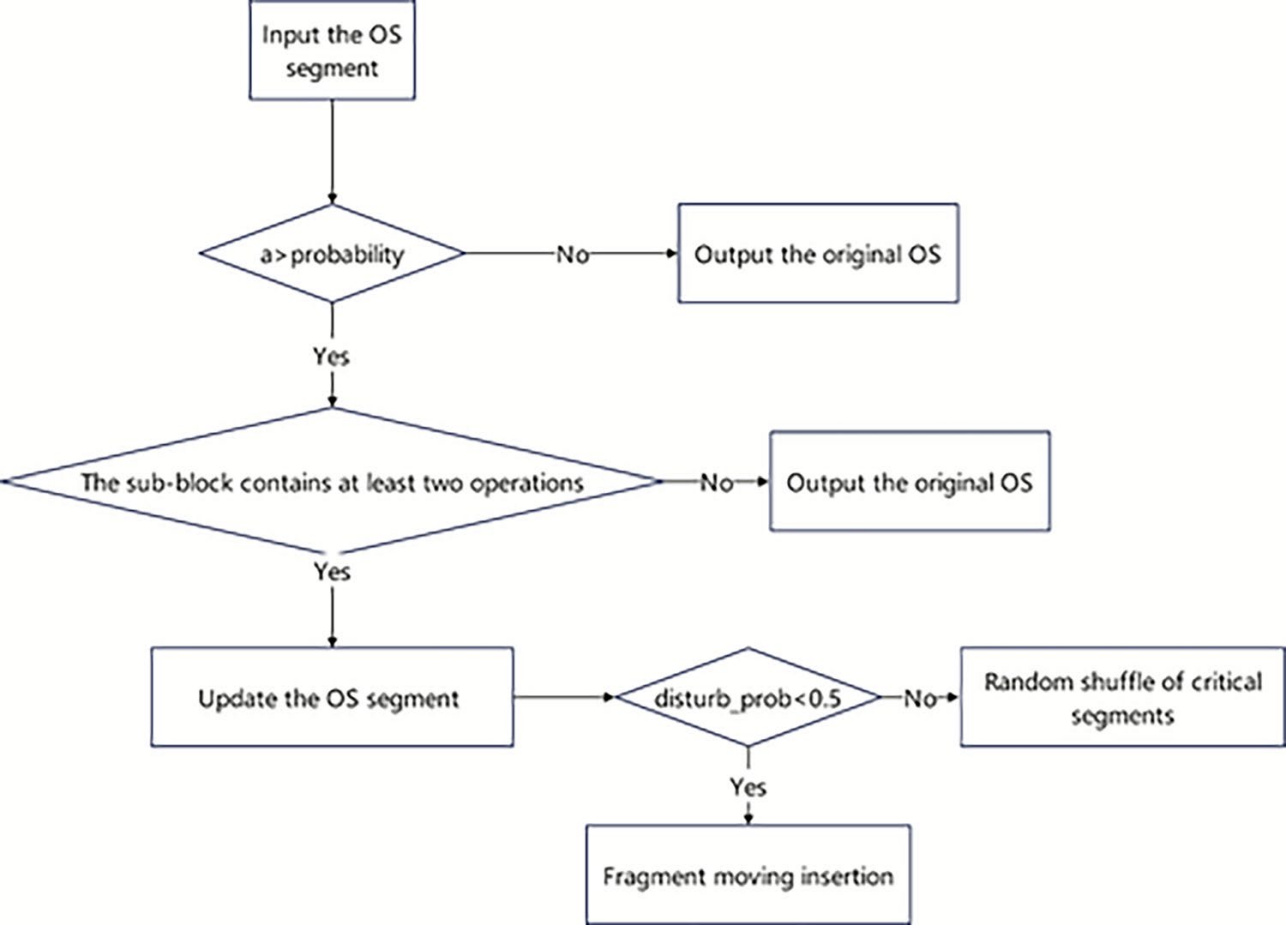
Flowchart of the critical-path-guided local search for OS.

If the sub-block length is at least 2, a critical-path-guided local search can be applied, the critical-path-guided local search for OS is illustrated in Figure 7.

1. Fragment moving insertion: In the identified sub-block, a continuous operation sequence fragment is intercepted. The length of the fragment is chosen randomly but is constrained to be at least 2 and at most the total number of operations in the block. The fragment is removed from its original position and inserted into another position inside the sub-block without affecting other unrelated operations. Before insertion, a section of the original content of the same length as the segment will be cleared at the target position to make room for the new fragment and avoid abnormalities in the updated solution.
2. Random shuffle of critical segments: This strategy performs random shuffling operations on all operations of the critical path block, generating a new local operation sequence. Such randomness enables the algorithm to escape a local optimum by introducing stronger perturbations. Compared with “fragment insertion”, random shuffling introduces greater disturbance, expands the search radius, increases population diversity, and enhances the algorithm’s global exploration ability.

For the machine selection, the information from the three leader wolves is utilized to guide the ordinary individuals to update the machine assignments for each operation. First, the machine selection segments of three leader wolves are extracted, and roulette wheel selection is used to select one from the three candidate machine values, which then serves as the new machine assignment for the corresponding operation. Specifically, the roulette wheel assigns fixed probabilities of 0.4, 0.3, and 0.3 to $\alpha$, $\beta$, and $\delta$, respectively, giving higher guidance weight to the best wolf while preserving diversity in machine assignment.

This method retains the guidance strategy under the traditional GWO framework, provides multi-source information as reference, and achieves more stable convergence.

### Adaptive mutation strategy

**Fig. 8:**
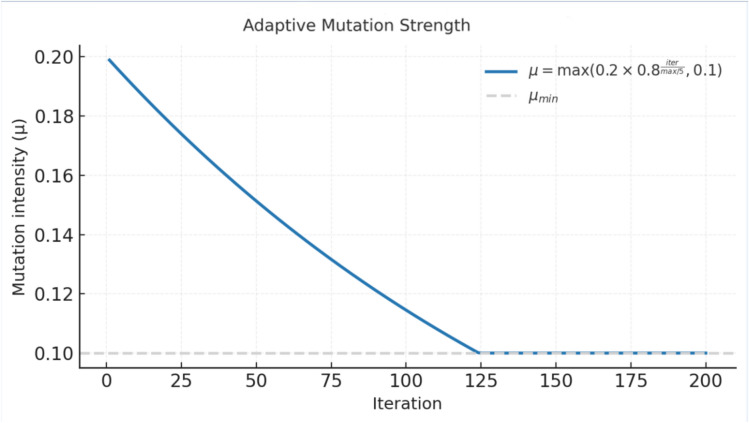
Variation curve of mutation strength $\mu$.

To enhance the adaptability of the algorithm in later search stages, an adaptive mutation operator from the genetic algorithm is incorporated into the proposed hybrid GWO framework. The mutation mechanism is well-structured and introduces only mild perturbations, it can be applied after the position update. To control the degree of disturbance over time, an adaptive exponential decay scheme is designed for the mutation intensity. The current mutation strength $\mu$ is as follows:

26 $$\begin{aligned} \mu =\max \left( \mu _{0}\cdot 0.8^{\frac{\text {iter}}{\text {max\_iter}/5}},\mu _{\text {min}}\right) \end{aligned}$$

where $\mu _{0}$ denotes the initial mutation intensity, which is set to 0.2 in this study; $\mu _\text {min}$ is the minimum mutation intensity, used to prevent premature stagnation, *iter* and $max\_iter$ are the current number of iterations and the maximum number of iterations, respectively. Following commonly used settings in adaptive metaheuristic frameworks^30^, we select 0.8 and 5 to maintain a moderate and steady decrease in mutation intensity over the course of the iterations , as shown in Figure 8.

Operation sequence mutation: For the operation sequence segment, a random swap mutation strategy is employed. Randomly select two positions from the current operation sequence and exchange them. This is a micro-perturbation operation, which can strengthen neighborhood exploration and facilitate local improvement.

Machine selection mutation: With a certain probability (mutation intensity), each gene in the machine selection sequence is independently perturbed. Specifically, for the operation at the selected mutation position, a new machine is randomly chosen from its corresponding set of available machines, replacing the original value, so as to result in a new machine assignment scheme.

The distinct feature of HGWO-DPDS lies in the synergy of three complementary strategies. The Discrete Prism Dispersion Strategy enhances population diversity and avoids premature convergence; the Position Update intensifies local search around the bottleneck operations that determine the makespan; and the Adaptive Mutation dynamically fine-tunes promising solutions in the later iterations. Working together, these three mechanisms balance global exploration and local exploitation, which makes HGWO-DPDS more robust and effective than variants using only one or two of them.

### Convergence and complexity assessment

From a theoretical perspective, although the proposed algorithm is essentially heuristic and does not guarantee strict mathematical convergence, its iterative process can be interpreted as a gradual contraction of the search toward high-quality regions. This provides a heuristic form of convergence in probability, consistent with the behavior observed in other swarm-based algorithms.

In terms of computational complexity, assuming the population size is *N*, the number of operations per iteration mainly depends on the decoding and fitness evaluation processes, resulting in an overall time complexity of $O(N \times n_{op} \times m)$, where $n_{op}$ and *m* indicate the number of operations and machines.

Table 4 summarizes the time complexity of each key component of HGWO-DPDS. Overall, the complexity of the proposed algorithm is comparable to that of other population-based metaheuristics such as GA and PSO, indicating that the proposed HGWO-DPDS does not incur significant additional computational overhead.

**Table 4:** Time complexity of each component.

Algorithm component	Time complexity
Initialization	$O(N \times n_{op})$
Decoding	$O(N \times n_{op} \times m)$
DPDS	$O(N \times n_{op})$
Position Update	$O(N \times n_{op} \times m)$
Adaptive Mutation	$O(N \times n_{op})$

## Experiments and discussion

To verify the effectiveness of the proposed hybrid grey wolf optimization algorithm based on a discrete prism dispersion strategy for solving the FJSP, comparative experiments are conducted on several benchmark datasets, including three widely used benchmark datasets: MK instances, Kacem instances, and Lawrence instances.

The MK instances were proposed by Brandimarte in 1993 as benchmarks for FJSP. They include 10 instances, with 10, 15, or 20 jobs, and 6, 8, 10, or 15 machines. Each job contains between 4 and 8 operations. Owing to their moderate size and structural diversity, MK instances have been widely adopted in the literature^31^. The Kacem instances were introduced by Kacem in 2002 for FJSP. There are four instances in total^32^, with scales of $4 \times 5$, $10 \times 7$, $10 \times 10$, and $15 \times 10$. Each instance contains approximately 1.5 to 2 times the number of jobs in operations. These instances are characterized by small alternative machine sets and clear processing time distributions, making them suitable for testing the capability of handling flexible processes. The Lawrence (LA) instances were initially proposed by Lawrence in 1984 for the classical job-shop scheduling problem (JSP)^33^, and have since been widely extended into flexible variants for testing FJSP algorithms. A total of 40 instances are included, with scales ranging from $10 \times 5$ to $30 \times 15$. In most cases, the number of operations is 2–3 times the number of jobs. These instances have higher complexity and larger solution spaces, making them well-suited for evaluating the performance of algorithms in large-scale scheduling scenarios involving multiple operations. They are also recognized as one of the most important benchmarks in FJSP research.

All experiments were conducted using Python, on a computer with Windows 11, a $12^{th}$ Gen Intel(R) Core(TM) i7-12700 2.10 GHz processor, and 8 GB of RAM. Each instance was run independently 20 times, and the results were evaluated based on the best makespan obtained and the relative deviation $(GAP\%)$.

### Experimental design

To ensure that the proposed algorithm maintains stable optimization performance across different instance scales, a systematic parameter sensitivity analysis was conducted. Considering the number of parameters and their complex interactions, the Taguchi orthogonal experimental design was adopted to improve experimental efficiency. By constructing an orthogonal array, the influence of each parameter on algorithm performance can be obtained with a limited number of experimental combinations, thereby determining a reasonable parameter configuration.

In the prismatic dispersion stage, the perturbation strength is defined as $w = \min (k \cdot \text {rand}(), 1)$. To improve reproducibility, the refraction array *k* was fixed at 0.5, 1.0, 1.5. Preliminary tests showed that this setting provides sufficient scaling diversity. Consequently, *k* was excluded from the subsequent parameter sensitivity analysis.

**Table 5:** Parameter settings for small-scales.

Parameter	Level 1	Level 2	Level 3
*N*	50	100	200
*T*	400	450	500
*r*	0.3	0.4	0.5
$\mu _0$	0.2	0.3	0.4

**Table 6:** Parameter settings for large-scales.

Parameter	Level 1	Level 2	Level 3
*N*	50	100	200
*T*	800	1000	1200
*r*	0.3	0.4	0.5
$\mu _0$	0.2	0.3	0.4

Four primary control parameters were considered for the algorithm. *N*: initial population size; *T*: maximum number of iterations; *r*: perturbation ratio of the critical path; $\mu _0$: initial value of the adaptive mutation strength.

Each parameter was assigned three levels, as shown in Tables 5 and 6, corresponding to the experimental settings for small-scale and large-scale instances, respectively. The small-scale experiments used MK01 and MK03 as representative instances, while MK07 and MK08 were selected for the large-scale experiments. An $L_9$ orthogonal array was employed to generate nine parameter combinations. Each experiment was repeated 20 times independently, and the average makespan (*Mean*), variance (*Var*), and runtime (*Time*) were recorded as performance indicators.

The experimental results are presented in Tables 7 and 8. For these instances, the performance variations due to different parameter levels are relatively small. Among the four parameters, the population size *N* and the perturbation ratio *r* have a slightly stronger influence on the optimization results, while *T* and $\mu _0$ exhibit minimal impact. For Table 7, the baseline times of the first combination are all below 1min, while for Table 8 they are below 3min. Although the computational time is influenced by population size, iteration number, and software environment, the differences among parameter combinations remain moderate, and the total running time is acceptable.

Overall, the average makespan differences remain moderate, indicating that the proposed algorithm maintains relatively stable performance across different parameter settings and problem scales.

**Table 7:** Orthogonal experiment for MK01 and MK03.

Number	*N*	*T*	*r*	$\mu _0$	MK01			MK03		
					Mean	Var	Time	Mean	Var	Time
1	50	400	0.3	0.2	43.2	0.98	1.00	218.4	5.44	1.00
2	50	450	0.4	0.3	43.45	1.12	1.25	215.4	4.76	1.16
3	50	500	0.5	0.4	43.6	1.16	1.02	216.3	4.92	1.64
4	100	400	0.4	0.4	43.3	1.14	1.29	216.9	5.39	2.14
5	100	450	0.5	0.2	42.9	0.89	1.69	215.7	5.68	2.81
6	100	500	0.3	0.3	43.6	0.92	1.59	214.0	4.82	2.36
7	200	400	0.5	0.3	42.8	1.15	2.67	213.2	2.04	4.37
8	200	450	0.3	0.4	43.6	0.92	4.06	214.5	5.68	4.13
9	200	500	0.4	0.2	43.2	1.08	4.53	215.00	4.00	4.41

**Table 8:** Orthogonal experiment for MK07 and MK08.

Number	*N*	*T*	*r*	$\mu _0$	MK07			MK08		
					Mean	Var	Time	Mean	Var	Time
1	50	800	0.3	0.2	159.5	3.67	1.00	528.2	5	1.00
2	50	1000	0.4	0.3	161.8	5.23	1.04	525.7	3.44	1.20
3	50	1200	0.5	0.4	159.3	3.74	0.97	523.7	1.27	1.45
4	100	800	0.4	0.4	157.2	3.19	1.23	524.5	1.63	1.56
5	100	1000	0.5	0.2	157	3.26	2.20	524.9	2.17	2.35
6	100	1200	0.3	0.3	155.8	3.31	1.71	524.3	1.55	2.19
7	200	800	0.5	0.3	157.9	4.32	2.70	524	1.10	3.84
8	200	1000	0.3	0.4	157.1	2.07	4.23	523.4	0.92	3.66
9	200	1200	0.4	0.2	155.7	2.22	4.78	523.9	0.40	4.60

The times are reported as relative values with respect to the first parameter combination of each instance (first row = 1.00) to better illustrate the trend.

For small-scale instances, configuration 7 ($N = 200$, $T = 400$, $r = 0.5$, $\mu _0 = 0.3$) not only achieves the lowest mean and relatively small variance, but also demonstrates good stability across instances; for large-scale instances, combinations with $N = 200$ generally show stable performance and low mean values. Further analysis indicates that the population size and maximum number of iterations have the most significant impact on performance, whereas adjustments to *r* and $\mu _0$ have minor effect. Therefore, configuration 7 is selected as the default parameter setting for small-scale problems, while configurations 8 or 9 are recommended for large-scale problems.

### Effectiveness analysis of HGWO-DPDS

To evaluate the effectiveness of the key improvement strategies proposed in this study, an ablation study was conducted by sequentially removing three core components from the full HGWO-DPDS framework and evaluating the performance, as shown in Table 9.

The ablated components include:

1. The discrete prism dispersion strategy,
2. The critical-path-based position update strategy,
3. The adaptive mutation operator.

Several algorithm variants were constructed for comparison:

1. GWO: the original grey wolf optimizer without enhancements, retaining only the standard leader-guided position update and integer encoding
2. PDGWO: removing the adaptive mutation operator while retaining the position update and dispersion strategy
3. PGWO: removing both the mutation and dispersion components, retaining only the improved position update mechanism

**Table 9:** Ablation study on MK instances.

Instance	Scale	Makespan Range	Optimal makespan			
			Our	GWO	PGWO	PDGWO
MK01	10$\times$6	36–42	41	53	45	43
MK02	10$\times$6	24–32	28	45	40	31
MK03	15$\times$8	204–211	204	270	231	214
MK04	15$\times$8	48–81	67	94	84	78
MK05	15$\times$4	168–186	178	186	204	182
MK06	10$\times$15	33–86	74	111	98	79
MK07	20$\times$5	133–157	149	182	180	155
MK08	20$\times$10	523	523	566	562	523
MK09	20$\times$10	299–369	359	431	412	368
MK10	20$\times$15	165–296	269	348	339	336

Removing the dispersion strategy reduces population diversity and causes premature convergence, while removing mutation weakens the late-stage refinement ability, explaining its smaller but non-significant performance drop. The large gap between PGWO and the GWO further confirms that our position-update mechanism is essential for solving the FJSP.

Table 10 presents the results of Wilcoxon signed-rank^34^ tests among the four compared algorithms. The proposed algorithm (Our) achieves *p*-values below the 0.05 significance threshold when compared with all three benchmark methods (GWO, PGWO, and PDGWO). In particular, the differences between Our and GWO as well as PGWO are notable ($p<0.01$), indicating that the proposed method outperforms the conventional GWO and its basic variant in terms of makespan minimization. The comparison with PDGWO also yields a significant result ($p<0.05$), further demonstrating the robustness and superiority of the proposed approach in solving the FJSP.

**Table 10:** Wilcoxon signed-rank test results and significance levels ($$^*p<0.05$$, $$^{**}p<0.01$$).

Comparison	p-value	Significance
Our vs GWO	0.0020	**
Our vs PGWO	0.0020	**
Our vs PDGWO	0.0077	**
GWO vs PGWO	0.0471	*
PGWO vs PDGWO	0.0020	**

Moreover, the comparisons between GWO and PGWO, and between PGWO and PDGWO, also indicate that the choice of improvement strategies can influence algorithm performance. Overall, the Wilcoxon test results confirm that the proposed algorithm achieves a consistent advantage over the compared methods in the considered benchmark instances.

To further highlight the performance differences among algorithms, we apply the Friedman test. The test results, $Chi-square statistic = 28.63$ and $p = 3 \times 10^{-6}$, indicating a statistically significant difference among the four algorithms. Also, as shown in Table 11, the Nemenyi test further shows the algorithm significantly outperforms both GWO and PGWO, with rank differences exceeding the critical difference (CD), which is 1.48. The CD is the minimum rank gap required for two algorithms to be considered significantly different. The difference between Our and PDGWO does not exceed the CD, suggesting that the performance improvement over PDGWO is not statistically significant. The small rank differences between Our vs PDGWO, GWO vs PGWO and PGWO vs PDGWO indicate that the incremental effects of proposed strategies.

**Table 11:** Nemenyi pairwise differences.

Comparison	Rank difference
Our vs GWO	2.85
Our vs PGWO	2.05
Our vs PDGWO	0.90
GWO vs PGWO	0.80
PGWO vs PDGWO	1.15

Overall, these results confirm the stability and effectiveness of the proposed algorithm.

**Fig. 9:**
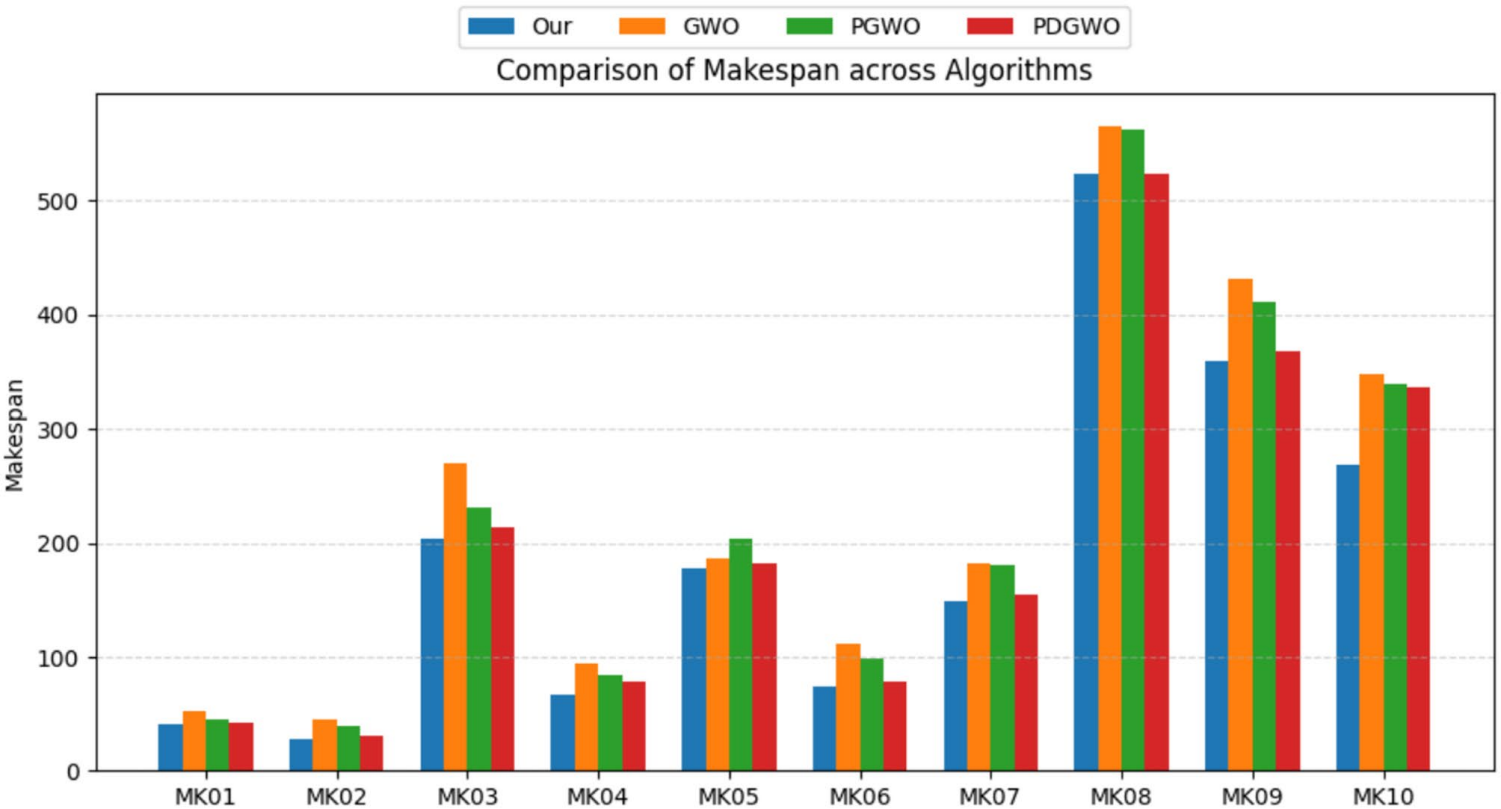
Comparison of makespan across instances.

Figure 9 provides a comparison of the makespan results obtained by the four algorithms across the ten MK instances. Overall, the proposed HGWO-DPDS algorithm (Our) achieves the best performance on most instances, as reflected by the lowest bar heights, with the advantage becoming more apparent for larger-scale problems. Compared with the original GWO, the PGWO and PDGWO algorithms, which progressively incorporate improvement strategies, exhibit a gradual enhancement in performance.

**Fig. 10:**
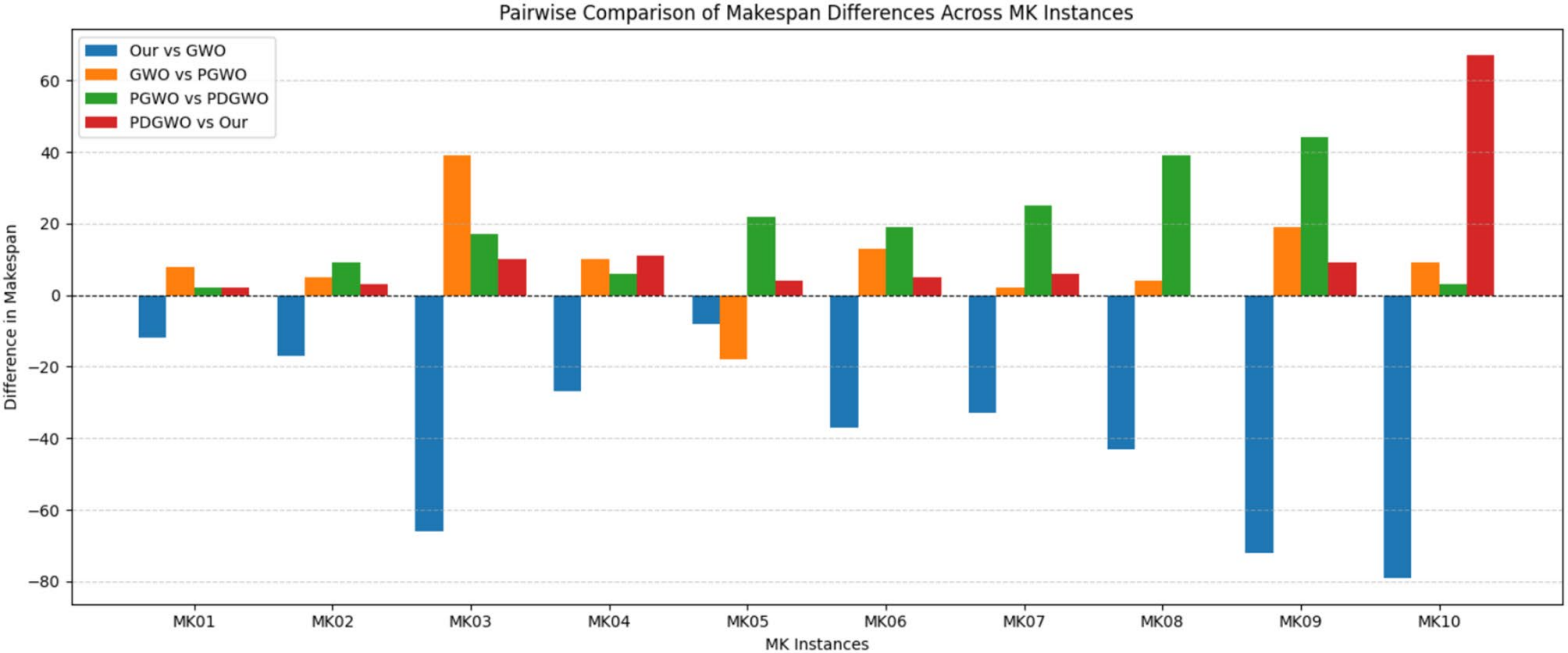
Pairwise comparison of makespan differences.

Figure 10 illustrates the pairwise differences in makespan among the algorithms, offering another perspective to confirm the above observations. The negative values of “Our vs GWO” across all instances indicate that HGWO-DPDS consistently outperforms the original GWO. The negative values of “PGWO vs PDGWO” suggest that the introduction of the discrete prism dispersion strategy effectively improves algorithmic performance. Furthermore, the negative “PDGWO vs Our” values imply that the complete HGWO-DPDS framework still maintains certain performance advantages over the PDGWO variant without the adaptive mutation operator. Together, these two figures provide clear supporting the effectiveness of the proposed strategies.

### Comparison with other algorithms

To further assess the effectiveness and competitiveness of the proposed algorithm, several representative algorithms from existing literature were selected for comparison. The comparison was first carried out on the MK and Kacem benchmark instances. The compared methods include traditional metaheuristic algorithms such as the Artificial Bee Colony (ABC), Genetic Algorithm (GA), and Particle Swarm Optimization (PSO)^35^, which have been widely applied and proven effective in early scheduling research. Additionally, several improved or hybrid algorithms were included, including the Improved Particle Swarm Optimization (IPSO)^36^, Hybrid Grey Wolf Optimizer (HGWO)^18^, Hybrid Whale Optimization Algorithm (HWOA)^37^, PSO with Tabu Search (PSO+TS)^38^, Improved Shuffled Frog Leaping Algorithm with Unified Gravitational Search (SFLA-uGSA)^39^, Deep Reinforcement Learning framework with a lightweight Multi-Layer Perceptron (DRL)^40^, the Deep Reinforcement Learning-based Dueling DQN (D5QN)^41^, and Heterogeneity-Enhanced Graph Neural Network Algorithm combined with Deep Reinforcement Learning (HGNN)^42^.

Tables 12 and 13 summarize the performance of HGWO-DPDS on the MK and Kacem instances.

**Table 12:** Scheduling results on MK instances.

Instance	Scale	Optimal range	ABC	GA	IPSO	PSO	HGWO	HWOA	DRL	D5QN	HGNN	Our
MK01	10$\times$6	36–42	42	42	41	42	41	41	44	42	52	41
MK02	10$\times$6	24–32	32	34	30	28	26	30	31	31	34	28
MK03	15$\times$8	204–211	213	213	204	204	207	204	211	204	212	204
MK04	15$\times$8	48–81	74	69	69	75	65	67	78	69	73	67
MK05	15$\times$4	168–186	185	183	178	179	171	171	183	180	182	178
MK06	10$\times$15	33–86	82	83	82	69	61	72	74	68	90	74
MK07	20$\times$5	133–157	165	168	147	149	173	149	156	153	199	149
MK08	20$\times$10	523	532	523	523	555	523	523	524	523	528	523
MK09	20$\times$10	299–369	386	344	339	342	307	321	326	315	340	359
MK10	20$\times$15	165–296	293	268	258	242	312	253	241	182	263	269

**Table 13:** Scheduling results on Kacem instances.

Instance	Scale	LB	GA	PSO+TS	ABC	GWO	SFLA-uGSA	Our
Kacem01	4$\times$5	11	12	11	11	12	11	11
Kacem02	10$\times$7	11	11	–	11	11	11	11
Kacem03	10$\times$10	7	10	8	9	10	7	9
Kacem04	15$\times$10	11	17	11	16	15	12	14

For ABC, GA and GWO, we re-implemented the algorithms according to the parameter settings reported in their original papers and executed them under exactly the same experimental conditions as the proposed method. For the remaining algorithms, we used the best results reported in the original literature for comparison.

Overall, the algorithm achieved the best-known solution in 4 instances and solutions within the known optimal bounds in 6 instances, demonstrating overall stable performance. Specifically, the algorithm attained the lower bound (LB) in MK03 and MK08. In medium-sized problems such as MK04, MK05, MK07, and MK10, the proposed method outperformed most existing approaches. However, in more challenging and highly discrete instances like MK09 and MK10, the performance was slightly less competitive. In the Kacem instances, the algorithm matched the optimal value in Kacem01 and Kacem02, and produced slightly higher makespan in Kacem03 and Kacem04. Overall, the algorithm maintained relatively small deviations across most instances.

Table 14 reports the scheduling results of the proposed HGWO-DPDS algorithm on a total of 40 standard LA instances $(LA01\text {--}LA40)$, and compares them with several representative algorithms from the literature, including the Hybrid Differential Evolution and Estimation of Distribution Algorithm based on Neighbourhood Search (NS-HDE/EDA)^43^, Improved Grey Wolf Optimizer (IGWO)^17^, Bacterial Foraging Optimization Algorithm (BFO)^44^, a deep reinforcement learning approach (DQN)^45^, a hybrid Genetic Simulated Annealing and Variable Neighborhood Search (GASAVNS)^12^, and a heuristic method based on the MWKR+SPT dispatching rule^46^.

**Table 14:** Scheduling results on LA instances.

Instance	Scale	LB	NS-HDE/EDA	IGWO	BFO	MWKR+SPT	DQN	GASAVNS	Our
LA01	10$\times$5	570	666	666	772	835	666	666	587
LA02	10$\times$5	529	655	655	661	853	655	655	553
LA03	10$\times$5	477	597	597	621	656	597	606	502
LA04	10$\times$5	502	590	590	594	681	609	609	531
LA05	10$\times$5	457	593	593	593	689	593	593	478
LA06	15$\times$5	799	926	926	987	1196	926	926	830
LA07	15$\times$5	749	890	890	902	893	890	890	756
LA08	15$\times$5	765	863	863	863	1055	863	863	775
LA09	15$\times$5	853	951	951	998	1077	951	951	861
LA10	15$\times$5	804	958	958	990	1101	958	958	815
LA11	20$\times$5	1071	1222	1222	1320	1429	1222	1222	1078
LA12	20$\times$5	936	1039	1039	1051	1235	1047	1039	940
LA13	20$\times$5	1038	1150	1150	1227	1249	1151	1150	1040
LA14	20$\times$5	1070	1292	1292	1292	1258	1292	1292	1078
LA15	20$\times$5	1089	1207	1207	1231	1387	1221	1219	1101
LA16	10$\times$10	717	956	956	965	1139	980	1000	795
LA17	10$\times$10	646	784	790	801	970	799	794	694
LA18	10$\times$10	663	855	859	977	1132	859	859	764
LA19	10$\times$10	617	852	845	871	978	872	860	803
LA20	10$\times$10	756	907	937	951	1029	924	924	824
LA21	15$\times$10	804	1058	1090	–	1403	1162	1132	1032
LA22	15$\times$10	736	952	970	–	1200	1021	1000	925
LA23	15$\times$10	815	1038	1032	–	1287	1053	1034	1023
LA24	15$\times$10	775	973	982	–	1326	1029	1000	987
LA25	15$\times$10	756	1000	1008	–	1370	1067	1061	984
LA26	20$\times$10	1054	1229	1239	–	1611	1327	1277	1255
LA27	20$\times$10	1084	1287	1290	–	1754	1397	1345	1317
LA28	20$\times$10	1070	1275	1263	–	1596	1386	1305	1267
LA29	20$\times$10	994	1220	1244	–	1643	1323	1290	1201
LA30	20$\times$10	1069	1371	1355	–	1759	1417	1370	1315
LA31	30$\times$10	1520	1784	1784	–	2081	1854	1784	1736
LA32	30$\times$10	1658	1850	1850	–	2277	1900	1850	1897
LA33	30$\times$10	1497	1719	1719	–	2305	1782	1719	1696
LA34	30$\times$10	1537	1721	1721	–	2248	1880	1748	1768
LA35	30$\times$10	1549	1888	1888	–	2098	1941	1888	1766
LA36	15$\times$15	948	1315	1311	–	1600	1355	1395	1295
LA37	15$\times$15	986	–	–	–	1700	1540	1504	1392
LA38	15$\times$15	943	–	–	–	1466	1348	1392	1255
LA39	15$\times$15	922	–	–	–	1679	1357	1281	1286
LA40	15$\times$15	955	–	–	–	1584	1336	1300	1271

From the experimental results, it can be observed that HGWO-DPDS achieved competitive scheduling performance in 35 out of the 40 instances. In the remaining cases, its results were only slightly worse than the best-performing comparative methods, while outperforming others, thereby demonstrating strong optimization capability. The algorithm performed well on small and medium-scale problems (such as $(LA01\text {--}LA05)$ and $(LA17\text {--}LA20)$), highlighting its effectiveness in local exploitation. For larger and more complex instances (such as LA28 and LA34), although the proposed approach did not always attain the best-known results, the performance gap remained narrow. This indicates that the critical-path-based perturbation mechanism and the prism dispersion strategy introduced in this work maintain robust adaptability and convergence performance in large-scale, high-complexity scheduling environments.

**Fig. 11:**
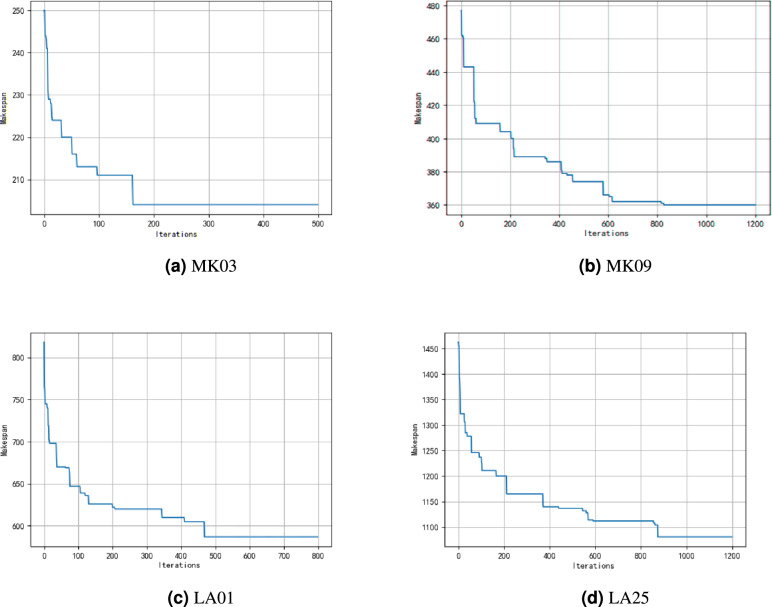
Representative convergence curves of HGWO-DPDS on selected instances.

To investigate the convergence behavior of the proposed algorithm on this set of instances, several representative convergence curves are illustrated in Figure 11, intuitively demonstrating the convergence effectiveness of the proposed algorithm within a reasonable number of iteration. As shown, for MK03, MK09, LA01, and LA25 instances, the algorithm exhibits a sharp decline in makespan during the initial iterations, reflecting its strong global exploration capability and the effectiveness in escaping from poor initial solutions. The convergence curves gradually flatten, indicating that the population approaches the optimal region and transitions to local exploitation.

However, it is also worth noting that the convergence speed decreases in the later stages, especially when approaching near-optimal solutions, where the improvement becomes marginal. This implies that there is still room for further enhancement in fine-grained local search strategies. Moreover, for some large-scale instances, the final solutions still exhibit a certain gap from the theoretical lower bounds, indicating that the exploitation capbility in complex solution spaces can be further improved.

This phenomenon can be mainly attributed to the expansion of the search space and intensified interaction complexity between operations and machines, which make it difficult to maintain convergence stability. Moreover, the discrete mapping and decoding process require significantly higher computational costs in large-scale problems, which may limit the algorithm’s exploration depth within a fixed iteration budget.

To quantitatively evaluate the optimization performance of the algorithm on the FJSP, *GAP* is adopted as one of the key performance metrics. It measures the deviation between the solution obtained by the algorithm and the optimal value or the theoretical lower bound of the given instance^46^. The (*GAP*) is defined as follows:

27 $$\begin{aligned} \text {GAP}(\%) = \frac{C_{\text {0}} - C_{\text {ref}}}{C_{\text {ref}}} \times 100\% \end{aligned}$$

where $C_{0}$ denotes the makespan obtained by the algorithm on a specific instance, and $C_{ref}$ refers to the corresponding reference value, which is either the theoretical lower bound (LB) or the best-known solution reported in the literature. A smaller *GAP* value indicates better scheduling performance on the instance, implying that the solution is closer to the optimum. As presented in Tables 15, 16, and 17, the *GAP* values for the three benchmark datasets are presented. It can be observed that HGWO-DPDS outperforms other methods on most instances in terms of *GAP*. These findings demonstrate the efficacy of the proposed multi-strategy cooperative framework in improving solution quality and reducing deviation.

**Table 15:** GAP (%) results of different algorithms on MK instances.

Instance	Scale	LB	ABC	GA	IPSO	PSO	HGWO	HWOA	DRL	D5QN	HGNN	Our
MK01	10$\times$6	36	16.67	16.67	13.89	16.67	13.89	13.89	22.22	16.67	44.44	13.89
MK02	10$\times$6	24	33.33	41.67	25.00	16.67	8.33	25.00	29.17	29.17	41.67	16.67
MK03	15$\times$8	204	4.41	4.41	0.00	0.00	1.47	0.00	3.43	0.00	3.92	0.00
MK04	15$\times$8	48	54.17	43.75	43.75	56.25	35.42	39.58	62.50	43.75	52.08	39.58
MK05	15$\times$4	168	10.12	8.93	5.95	6.55	1.79	1.79	8.93	7.14	8.33	5.95
MK06	10$\times$15	33	148.48	151.52	148.48	109.09	85.45	118.18	124.24	106.06	172.73	124.24
MK07	20$\times$5	133	24.06	26.32	10.53	12.03	30.08	12.03	17.29	15.04	49.62	12.03
MK08	20$\times$10	523	1.72	0.00	0.00	6.12	0.00	0.00	0.19	0.00	0.96	0.00
MK09	20$\times$10	299	29.10	15.05	13.38	14.38	2.68	7.36	9.03	5.35	13.71	20.07
MK10	20$\times$15	165	77.58	62.42	56.36	46.67	89.09	53.33	46.06	10.30	59.39	63.03

**Table 16:** GAP(%) results on Kacem instances.

Instance	Scale	LB	GA	PSO+TS	ABC	GWO	SFLA-uGSA	Our
Kacem01	4$\times$5	11	9.09	0.00	0.00	9.09	0.00	0.00
Kacem02	10$\times$7	11	0.00	–	0.00	0.00	0.00	0.00
Kacem03	10$\times$10	7	14.29	14.29	28.57	42.86	0.00	28.57
Kacem04	15$\times$10	11	45.45	0.00	45.45	36.36	9.09	27.27

As shown in Tables 15 and 16, the proposed HGWO-DPDS demonstrates strong competitiveness on the MK and Kacem benchmark sets. On the MK instances, it achieves the smallest *GAP* values in more than half of the cases and reaches the theoretical lower bound in MK03 and MK08. The algorithm maintains moderate deviations on instances such as MK04 and MK05, outperforming most comparison methods. However, for larger and more complex cases such as MK09 and MK10, the deviation slightly increases, which can be attributed to the reduced effectiveness of the critical-path perturbation in high-dimensional search spaces. On the Kacem benchmarks, HGWO-DPDS attains optimal or near-optimal results in all four instances, reflecting its strong adaptability to problems with a limited number of machines.

**Table 17:** GAP(%) results on LA instances.

Instance	Scale	LB	NS-HDE/EDA	IGWO	BFO	MWKR+SPT	DQN	GASAVNS	Our
LA01	10$\times$5	570	16.84	16.84	35.44	46.49	16.84	16.84	2.98
LA02	10$\times$5	529	23.82	23.82	24.95	61.25	23.82	23.82	4.54
LA03	10$\times$5	477	25.16	25.16	30.19	37.53	25.16	27.04	5.24
LA04	10$\times$5	502	17.53	17.53	18.33	35.66	21.31	21.31	5.78
LA05	10$\times$5	457	29.76	29.76	29.76	50.77	29.76	29.76	4.60
LA06	15$\times$5	799	15.89	15.89	23.53	49.69	15.89	15.89	3.88
LA07	15$\times$5	749	18.83	18.83	20.43	19.23	18.83	18.83	0.93
LA08	15$\times$5	765	12.81	12.81	12.81	37.91	12.81	12.81	1.31
LA09	15$\times$5	853	11.49	11.49	17.00	26.26	11.49	11.49	0.94
LA10	15$\times$5	804	19.15	19.15	23.13	36.94	19.15	19.15	1.37
LA11	20$\times$5	1071	14.10	14.10	23.25	33.43	14.10	14.10	0.65
LA12	20$\times$5	936	11.00	11.00	12.29	31.94	11.86	11.00	0.43
LA13	20$\times$5	1038	10.79	10.79	18.21	20.33	10.89	10.79	0.19
LA14	20$\times$5	1070	20.75	20.75	20.75	17.57	20.75	20.75	0.75
LA15	20$\times$5	1089	10.84	10.84	13.04	27.36	12.12	11.94	1.10
LA16	10$\times$10	717	33.33	33.33	34.59	58.86	36.68	39.47	10.88
LA17	10$\times$10	646	21.36	22.29	23.99	50.15	23.68	22.91	7.43
LA18	10$\times$10	663	28.96	29.56	47.36	70.74	29.56	29.56	15.23
LA19	10$\times$10	617	38.09	36.95	41.17	58.51	41.33	39.38	30.15
LA20	10$\times$10	756	19.97	23.94	25.79	36.11	22.22	22.22	8.99
LA21	15$\times$10	804	31.59	35.57	–	74.50	44.53	40.80	28.36
LA22	15$\times$10	736	29.35	31.79	–	63.04	38.72	35.87	25.68
LA23	15$\times$10	815	27.36	26.63	–	57.91	29.20	26.87	25.52
LA24	15$\times$10	775	25.55	26.71	–	71.10	32.77	29.03	27.35
LA25	15$\times$10	756	32.28	33.33	–	81.22	41.14	40.34	30.16
LA26	20$\times$10	1054	16.60	17.55	–	52.85	25.90	21.16	19.07
LA27	20$\times$10	1084	18.73	19.00	–	61.81	28.87	24.08	21.49
LA28	20$\times$10	1070	19.16	18.04	–	49.16	29.53	21.96	18.41
LA29	20$\times$10	994	22.74	25.15	–	65.29	33.10	29.78	20.82
LA30	20$\times$10	1069	28.25	26.75	–	64.55	32.55	28.16	23.01
LA31	30$\times$10	1520	17.37	17.37	–	36.91	21.97	17.37	14.21
LA32	30$\times$10	1658	11.58	11.58	–	37.33	14.60	11.58	14.41
LA33	30$\times$10	1497	14.83	14.83	–	53.97	19.04	14.83	13.29
LA34	30$\times$10	1537	11.97	11.97	–	46.26	22.32	13.73	15.03
LA35	30$\times$10	1549	21.89	21.89	–	35.44	25.31	21.89	14.01
LA36	15$\times$15	948	38.71	38.29	–	68.78	42.93	47.15	36.60
LA37	15$\times$15	986	–	–	–	72.41	56.19	52.54	41.18
LA38	15$\times$15	943	–	–	–	55.46	42.95	47.61	33.09
LA39	15$\times$15	922	–	–	–	82.10	47.18	38.94	39.48
LA40	15$\times$15	955	–	–	–	36.13	65.86	39.90	33.09

In Table 17, HGWO-DPDS also demonstrates good scalability on the LA benchmark set. It achieves the lowest *GAP* values in most instances, with deviations generally below 10$\%$ for $LA01\text {--}LA15$ and remaining competitive in more complex cases such as $LA28\text {--}LA34$. In a few very complex instances $(LA37\text {--}LA40)$, the improvement margin slightly narrows.

**Fig. 12:**
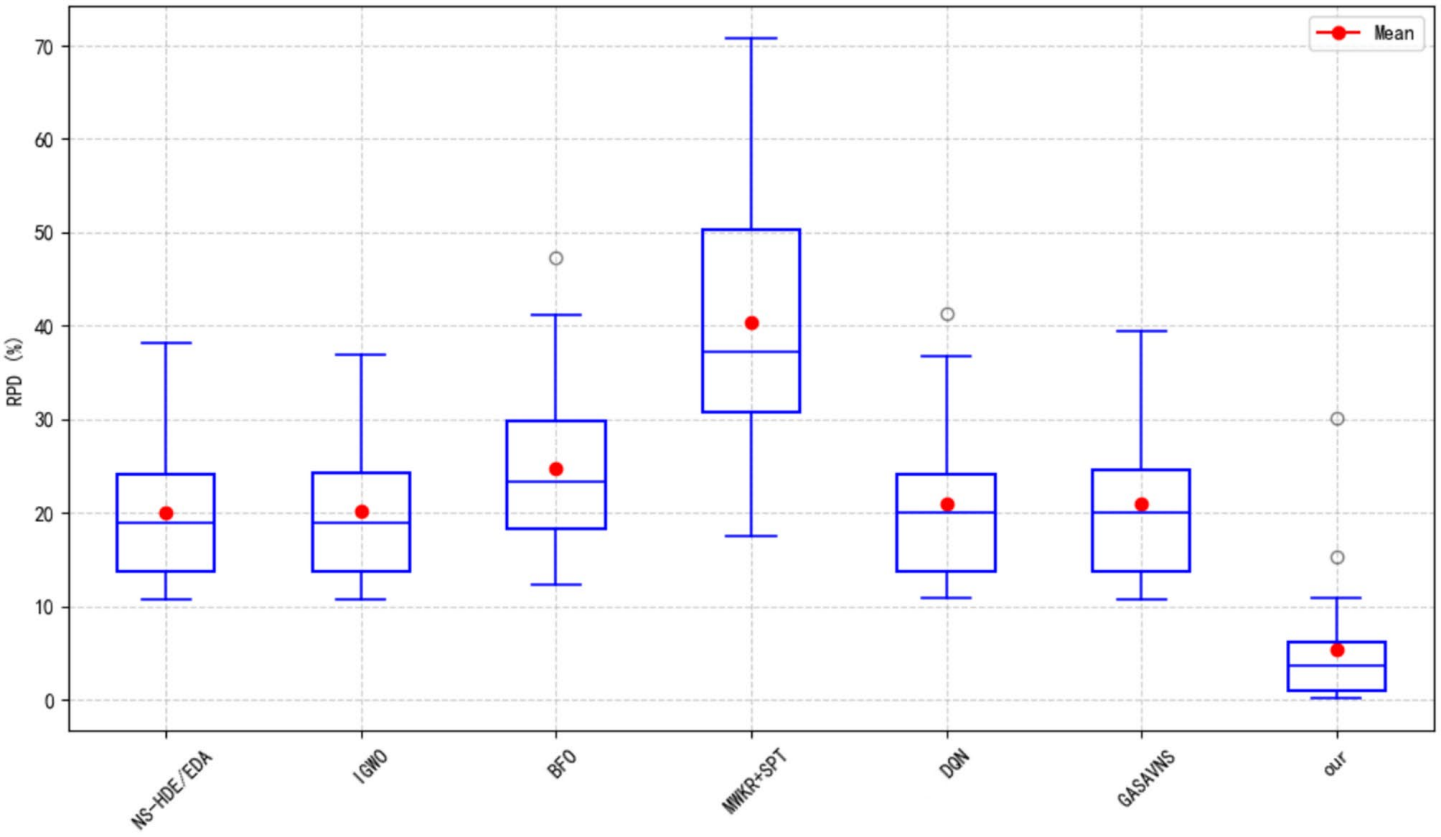
Boxplot of RPD values on LA instances.

To provide a more intuitive comparison of algorithmic performance on the LA dataset, Figure 12 illustrates the boxplots of Relative Percentage Deviation (RPD) values for all the methods across the 40 standard LA benchmark instances. In the figure, red dots indicate the average RPD for each algorithm, while the blue boxes represent the distribution and stability of the results.

As can be seen from the figure, the proposed HGWO-DPDS algorithm outperforms other methods in terms of both overall performance and stability. Specifically, it achieves the lowest average RPD, significantly lower than those of the six comparison algorithms, indicating that it consistently produces solutions closer to the lower bounds across most instances. In addition, the smallest box height indicates that the proposed algorithm achieves the narrowest interquartile range, reflecting highly consistent results with strong robustness and minimal performance fluctuation. In contrast, other methods such as MWKR+SPT and BFO exhibit several extreme outliers, reflecting unstable behavior. The MWKR+SPT rule, in particular, shows high dispersion with a maximum RPD approaching $70\%$, while HGWO-DPDS maintains its maximum RPD below $10\%$, further highlighting the applicability and superiority of the proposed hybrid optimization approach in solving complex scheduling problems.

Based on the experimental results, it can be concluded that the proposed HGWO-DPDS algorithm achieves optimal or near-optimal solutions in the majority of benchmark instances. It also exhibits stable average performance, with most *GAP* values lower than those of the comparison algorithms. In summary, these results further confirm that HGWO-DPDS provides high-quality solutions with strong consistency and adaptability across diverse flexible scheduling scenarios, making it a more reliable and robust optimization strategy for solving FJSP.

## Conclusion

This study proposes a hybrid Grey Wolf Optimization algorithm based on a Discrete Prism Dispersion Strategy (HGWO-DPDS), aiming to minimize the makespan in the FJSP. First, a feasible encoding and decoding scheme is designed. Second, a position update mechanism based on critical path perturbation is implemented, and multiple reference centers are introduced through the discrete prism dispersion strategy to expand the search space. In addition, an adaptive mutation operator is incorporated to enhance the adaptability and fine-tuning ability. Experimental results on public benchmark datasets show that HGWO-DPDS achieves high-quality scheduling performance on most instances. However, for some large-scale problems, the algorithm may require additional refinement in exploration or structural design to improve search efficiency and scalability.

Future research directions include:

1. Extending the algorithm to dynamic and uncertain manufacturing environments, such as machine breakdowns and varying job arrivals, to improve real-time adaptability and robustness.
2. Incorporating multi-objective optimization such as energy efficiency, machine load balance to enhance the practical applicability of the proposed algorithm.
3. Incorporating intelligent learning mechanisms such as deep reinforcement learning or self-adaptive parameter control to improve exploitation ability, decision-making accuracy, and performance on large-scale FJSP cases.

## Data Availability

The benchmark datasets used in this study, including the MK instances, Kacem instances, and Lawrence instances, are all publicly available. Datasets generated or analyzed during this study are available from the corresponding author upon reasonable request. Brandimarte (MK) instances were first introduced by Brandimarte^31^. Kacem instances were originally proposed by Kacem et al.^32^. Hurink instances were proposed by Hurink et al.^33^. Although the original articles did not include formal data availability statements, these datasets have since become standard benchmarks in flexible job-shop scheduling research. They are freely accessible from established public repositories, such as various open-source implementations on GitHub, and have been extensively adopted in subsequent studies. A minimal executable decoding script is available at https://github.com/fjspS/HGWO-DPDS. Additional data or analysis details will be made available upon request.
